# Welcome to the BSR Annual Meeting 2018

**DOI:** 10.5334/jbsr.1646

**Published:** 2018-11-17

**Authors:** Geert Villeirs

**Affiliations:** 1 UZ Gent, BE

Dear Colleagues,

Welcome to the Annual Meeting of the Belgian Society of Radiology.

This year’s edition will focus on **Interventional Radiology** and **Head & Neck Imaging**, featuring parallel sessions organized by the BSR Scientific Council and the Young Radiologist Section. National and international experts in the field will share their expertise and opinion on these topics in both cutting-edge lectures and refresher courses.

After lunchtime, a short **Plenary Session** will provide some background about the current status and future directions in Belgian radiology, directly followed by an exciting session on **Artificial Intelligence in Radiology**.

The formal program this time will be concluded with a sparkling **Social Event** with drinks, food and music organized by the Young Radiologist Section!

I gladly take the opportunity to particularly thank Professor Raymond Oyen, President of the Scientific Council, and Drs. Cedric Bohyn and Pierre-Antoine Poncelet, Presidents of the Young Radiologist Section, and all others involved in the organization of this Annual Meeting, for again a great job well done.

I wish you all a both interesting and pleasant BSR Annual Meeting 2018!

Warm regards,

Prof. Dr. Geert Villeirs

BSR President

